# Overexpression of *Pennisetum purpureum CCoAOMT* Contributes to Lignin Deposition and Drought Tolerance by Promoting the Accumulation of Flavonoids in Transgenic Tobacco

**DOI:** 10.3389/fpls.2022.884456

**Published:** 2022-05-10

**Authors:** Jian-Ling Song, Ze-Yu Wang, Yin-Hua Wang, Juan Du, Chen-Yu Wang, Xiang-Qian Zhang, Shu Chen, Xiao-Ling Huang, Xin-Ming Xie, Tian-Xiu Zhong

**Affiliations:** ^1^Office of Academic Research, Xingyi Normal University for Nationalities, Xingyi, China; ^2^Department of Grassland Science, College of Forestry and Landscape Architecture, South China Agricultural University, Guangzhou, China; ^3^Guangdong Engineering Research Center for Grassland Science, Guangzhou, China; ^4^Institute for Agricultural Biosciences, Oklahoma State University, Ardmore, OK, United States

**Keywords:** elephant grass, drought tolerance, *PpCCoAOMT*, lignin, flavonoids

## Abstract

Elephant grass (*Pennisetum purpureum*) is a fast-growing and low-nutrient demand plant that is widely used as a forage grass and potential energy crop in tropical and subtropical regions of Asia, Africa, and the United States. Transgenic tobacco with the *PpCCoAOMT* gene from *Pennisetum purpureum* produces high lignin content that is associated with drought tolerance in relation to lower accumulation of reactive oxygen species (ROS), along with higher antioxidant enzyme activities and osmotic adjustment. In this study, transgenic tobacco plants revealed no obvious cost to plant growth when expressing the *PpCCoAOMT* gene. Metabolomic studies demonstrated that tobacco plants tolerant to drought stress accumulated flavonoids under normal and drought conditions, which likely explains the observed tolerance phenotype in wild-type tobacco. Our results suggest that plants overexpressing *PpCCoAOMT* were better able to cope with water deficit than were wild-type controls; metabolic flux was redirected within primary and specialized metabolism to induce metabolites related to defense to drought stress. These results could help to develop drought-resistant plants for agriculture in the future.

## Key Message

- The *PpCCoAOMT* gene contributes to lignin deposition and flavonoid accumulation; it plays important roles in controlling drought tolerance, stomatal number, and aperture size without any plant growth cost.

## Introduction

The plant cell wall is the first barrier against external hazards, such as biotic and abiotic stressors; lignin is one of the most important functional components of the cell wall for multiple cells in plant tissues (Barros et al., 2015). As a major secondary metabolite, lignin is produced by the phenylpropanoid pathway. Lignin metabolism involves lignin biosynthesis, transport, and polymerization enzymes. Among the synthetic network of lignin monomers in the cytoplasm, caffeoyl-CoA O-methyltransferase (CCoAOMT) is a key enzyme that participates in the first methylation step required to produce G units, which are the three structural units (p-hydroxyphenyl, guaiacyl, and sinapyl moieties) from monolignols (Xia et al., 2018). In addition, CCoAOMT provides feruloyl-CoA for other biosynthetic pathways (Kai et al., 2008). The downregulation of *CCoAOMT* results in a decrease in lignin content (Wagner et al., 2011; Li et al., 2013), while the overexpression of *CCoAOMT* results in an increase in lignin content (Zhang et al., 2014; Zhao et al., 2021), indicating an essential role for CCoAOMT in lignin biosynthesis.

Lignin has many biological functions, such as water transport, mechanical support, and resistance to various stressors. Notably, low molecular weight lignin monomers derived from H and G units have antioxidant capacity; thus, lignin has some biological activity. Oxidative stress includes excessive production of reactive oxygen species (ROS), such as superoxide radicals (O_2_^–^) and hydrogen peroxide (H_2_O_2_), which are produced under adverse environmental conditions. H_2_O_2_ has an important regulatory role in lignin biosynthesis (Kováčik et al., 2010; Liu et al., 2015; Rui et al., 2016), indicating that the lignin biosynthetic pathway and the abiotic response may participate in crosstalk (Guo et al., 2017). A previous study demonstrated that *CCoAOMT* expression is enhanced under drought stress in the root elongation region of soybean (Yamaguchi et al., 2010), in grape berries (Giordano et al., 2016), and in the stem and leaf tissues of switchgrass (Liu et al., 2016). Many studies have shown that lignin biosynthesis is enhanced under drought stress and in response to other abiotic stressors. Lignin content significantly increases in the stem when plants are exposed to drought stress (Hu et al., 2009; Moura-Sobczak et al., 2011; Geng et al., 2018; Li et al., 2022). Moreover, an increase in lignin deposition is required for drought tolerance (Hu et al., 2009; Srivastava et al., 2015; Geng et al., 2018; Zhou et al., 2020) and the metal ion response (Xia et al., 2018; Su et al., 2020) *via* overexpression of lignin biosynthesis-related genes. For example, increased lignin content and improved drought tolerance are caused by the overexpression of genes such as *PoCCoAOMT* in *Nicotiana tabacum* (Zhao et al., 2021), *Gh4CL7* in *Arabidopsis thaliana* (Sun S. C. et al., 2020), *PtoMYB170* in *Populus tomentosa* (Xu et al., 2017), and *PuC3H35* in *Populus ussuriensi*s (Li et al., 2022).

Elephant grass (*Pennisetum purpureum*) is a fast-growing low nutrient demand plant that has potential for use in bio-oil production (Strezov et al., 2008) and for the alleviation of feed shortage because it is drought-resistant which could endure 5 mm monthly precipitation (Gomide et al., 2011) and produces high biomass (Rusdy, 2016). Elephant grass is a species of perennial C_4_ deep-rooted bunchgrass that inhabits tropical and subtropical regions of Asia, Africa, and the United States (Yan et al., 2021). The modulation of lignin deposition *via CCoAOMT* has been implicated in environmental stress responses in several species (Xia et al., 2018; Su et al., 2020; Zhao et al., 2021). However, few studies have explored the crosstalk between the lignin biosynthetic pathway and other biosynthetic pathways involved in drought tolerance. In the present study, we report that the overexpression of *PpCCoAOMT* in tobacco plants led to lignin accumulation but exhibited a contrasting effect on leaf area under transient drought conditions. The full-length cDNA of *P. purpureum CCoAOMT* was cloned and an overexpression construct of *CCoAOMT* (OE-*PpCCoAOM*T) was produced in transgenic tobacco plants by *Agrobacterium*-mediated transformation. The growth and development phenotypes of OE-*PpCCoAOMT* transgenic tobacco were evaluated and compared to wild-type (WT) plants. OE-*PpCCoAOMT* transgenic tobacco had higher drought stress tolerance because of ROS scavenging and higher maintenance of relative water content (RWC). Metabolomics analyses showed that *PpCCoAOMT* conferred transgenic tobacco with primary metabolites, resulting in more flavonoids that exhibited high antioxidant activities. These results will provide insights concerning the *CCoAOMT* lignin biosynthesis gene and the regulation of drought tolerance.

## Materials and Methods

### Isolation and *PpCCoAOMT* Bioinformatics Analysis

Total RNA of elephant grass (*P. purpureum*) was extracted from leaves and immediately reverse transcribed to obtain cDNA; the cDNA was used as a template to amplify the coding sequence of the *PpCCoAOMT* gene *via* 3′ random amplification of DNA ends (RACE) technology. Genomic DNA was used to amplify the 5′ end of the *PpCCoAOMT* gene using the high-efficiency thermal asymmetric interlaced polymerase chain reaction (hiTAIL-PCR). The PCR product was subjected to agarose gel electrophoresis and verified by sequencing. The homology of the PpCCoAOMT protein was analyzed by BLASTP. The amino acid sequences were aligned with DNAMAN software; conserved domains were identified using Red hollow boxes. Forty-one experimentally proven or putative CCoAOMT sequences from various species were used to construct a neighbor-joining phylogenetic tree in MEGA 6.0 software; 500 bootstrap replicates were used to estimate branch support.

### Verification of *PpCCoAOMT* Overexpressing Transgenic Tobacco Plants

*Agrobacterium* strain *LBA4404* harboring pBA002/3HA-*PpCCoAOMT* was transformed into tobacco (*N. tabacum* cultivar Wisconsin 38) using a leaf disc transformation method; regenerated shoots were rooted in the presence of 5 mg/L Basta before the plants were transferred to pots. The T0-generation of the transformed plants was identified by both PCR and RT-PCR. Total genomic DNA from all transgenic plants was extracted using the sodium dodecyl sulfate method. PCR was performed to confirm positive transformants using primers for the selectable marker gene BASTA (Bar: 5′-CGACTGCCAGAAACCCACGT-3′, 5′-CTGCACCATCGTCAACCACT-3′); the tobacco actin gene served as the internal control. After the removal of negatively transformed plants, the remaining plants were confirmed by specific primers. Total RNA was extracted from different OE plants and used for RT-PCR performed with a pair of primers (*PpCCoAOMT*: 5′-GGACATCAACCGCGAGAACT-3′, 5′-AGGTAGTTGTCCTTGTCGGC-3′) specific to the *PpCCoAOMT* gene. The tobacco actin gene was used as the internal control to normalize RNA expression (Cen et al., 2020).

### Stomatal Observations and Lignin Quantification

The adaxial side of the same position on the leaf was used for analyses of stomatal number, stomatal area, stomatal length, and stomatal width by means of the Scotch tape method. Photographs of stomata were captured using an advanced optical microscope (BX51) with a DP70 charge-coupled device camera (Olympus, Tokyo, Japan). Image-Pro Plus 6.0 software (Media Cybernetics, Silver Spring, MD, United States) was used to determine stomatal area, length, and width; a microscopic ruler (Olympus, Tokyo, Japan) was used to calibrate size.

The upper and middle internodes of the tobacco plants were embedded in 10% agarose (Sigma-Aldrich, St. Louis, MO, United States) and sectioned using a VT1000 vibratome (Leica, Sollentuna, Sweden). Transverse cross-sections were stained with Wiesner reagent and photographed using an Olympus BX51 microscope (Blaschek et al., 2020). The dye width was calculated using Image-Pro Plus 6.0 software. The lignin contents of the upper and middle internodes of tobacco were quantified in accordance with the method established by Li et al. (2011), which comprised a modified Klason method with three biological replicates. Statistical analyses were conducted using independent samples *t*-tests in SPSS 26.0 software (IBM Corp., Armonk, NY, United States).

### Drought Stress Treatment and Physiological Parameters

Six-week-old tobacco plants were grown in 1-gallon pots containing a mixture of potting soil and vermiculite (4:1, V/V) under natural conditions at the South China Agricultural University. During the experiment, daily maximum and minimum temperatures were 36 and 24°C. Daily maximum and minimum relative humidity was 82 and 78%. Moreover, a random block design was adopted using three replicates of each of the WT and OE plants. When the plants reached a height of 35 cm, they were subjected to drought stress *via* cessation of irrigation. The plant height, stem diameter, internode number, leaf area, leaf blade length, and leaf blade width were analyzed using Image-Pro Plus software. Leaf relative water content (RWC), proline content, malondialdehyde (MDA) content, and antioxidant enzyme activities were determined in accordance with methods described in our previous study (Zhong et al., 2014). H_2_O_2_ and O_2_ were detected using the 3,3-diaminobenzidine (DAB) and nitro blue tetrazolium (NBT) staining methods established by Wang et al. (2007). Analysis of variance was carried out on all data using SPSS software. *P*-values < 0.05 were considered statistically significant.

### Widely Targeted Metabolomics Analysis

Plant materials used for the metabolomics analysis were identical to the materials used for measurement of physiological indices after 0 and 17 days of drought treatment. Approximately 1.5 g of fresh sample were collected from all OE and WT plants, immediately frozen in liquid nitrogen, and stored at –80°C. Subsequently, these samples were sent to Wuhan Metware Biotechnology Co., Ltd. (Wuhan, China) for widely targeted metabolomics analysis. The sample extracts were analyzed using an ultrahigh performance liquid chromatography-electrospray ionization-tandem mass spectroscopy system (SHIMADZU Nexera X2^1^ ; Applied Biosystems 4500 Q TRAP).^2^

Unsupervised principal component analysis (PCA), hierarchical cluster analysis, and other multivariate statistical analysis methods were applied. The identified metabolites were annotated using the KEGG Compound database^3^ ; the annotated metabolites were mapped to the KEGG Pathway database.^4^ Pathways with significantly regulated metabolites were then fed into a metabolite set enrichment analysis; their significances were determined by hypergeometric test *p*-values.

## Results

### *PpCCoAOMT* Isolation and Genetic Analysis

The coding sequence of the *PpCCoAOMT* cDNA measured 801 bp, encoding a protein of 266 amino acid residues with a molecular weight of 29.7 KDa and a theoretical isoelectric point of 5.15; the length of the corresponding genomic sequence was 1,272 bp. Based on 3′ RACE and hiTAIL-PCR methods, the elephant grass genomic DNA template was amplified to obtain a sequence of 1,804 bp (Nucleotide Accession No. KJ9957361); the numbers of exons and introns, along with their lengths, were analyzed using Genescan software. The results showed that the *PpCCoAOMT* gene has five exons and four introns when applying the “GT-AG” rule, as well as a promoter region of 349 bp and a 3′ untranslated region (UTR) of 184 bp (Supplementary Figure 1).

**FIGURE 1:**
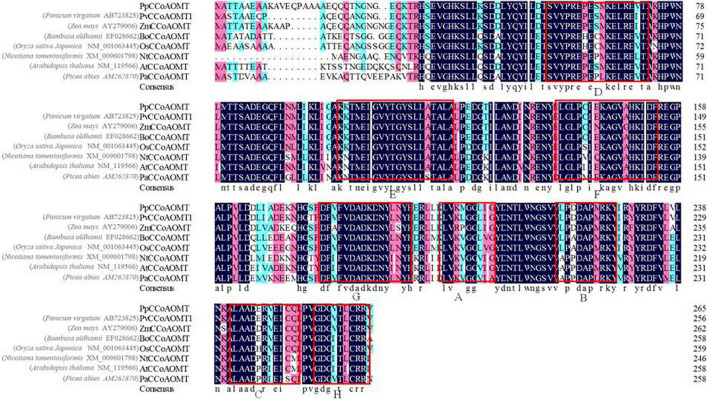
Multiple sequence alignment indicated that the CCoAOMT amino acid sequence of elephant grass (*Pennisetum purpureum*) contained motifs that were highly conserved in other species. Red hollow boxes indicate conserved motif patterns.

A BLASTP search showed that the PpCCoAOMT amino acid sequence shared 97% identity with a query cover of 92% from *Zea mays* (AAQ89900), 95% identity with a query cover of 100% from *Panicum virgatum* (AFY17068), and 90% identity with a query cover of 92% from *Bambusa oldhamii* (ABO26812). Subsequently, the neighbor-joining phylogenetic tree involving 41 orthologs was constructed using MEGA 6.0 after multiple alignments of the protein sequences using Clustal X, which demonstrated that PpCCoAOMT was closely grouped with monocot plant species; the highest homology was with the predicted protein of *Panicum virgatum* (Supplementary Figure 2). Seven amino acid sequences of known CCoAOMTs were selected based on evolutionary relationships and alignments with PpCCoAOMT. Although the length varied, eight conserved motifs were found in which A, B, and C methyl donor domains were ubiquitous characteristic elements; the D, E, F, G, and H domains represented specific conserved motifs for Mg^2+^ and substrate binding (Zhang Y. et al., 2019) in the CCoAOMT family (Figure 1).

### Overexpression of *PpCCoAOMT* Increases Lignin Content

The recombinant vector was prepared in pBA002/3HA under control of the CaMV35S promoter to upregulate *PpCCoAOMT* gene expression, then mobilized into *A. tumefaciens* strain *LBA4404* for genetic transformation of tobacco. PCR and RT-PCR were used together to identify the transgenic tobacco plants. PCR analysis demonstrated that all tobacco plants detected the NtActin product; one expected band of the selectable marker gene BASTA (Bar, 438 bp) was observed in the transgenic lines, whereas it was not observed in WT plants (Supplementary Figure 3A). Furthermore, RT-PCR results showed that the *PpCCoAOMT* gene was expressed in most transgenic plants, but the WT tobacco plants did not show any amplification (Supplementary Figure 3B).

**FIGURE 2:**
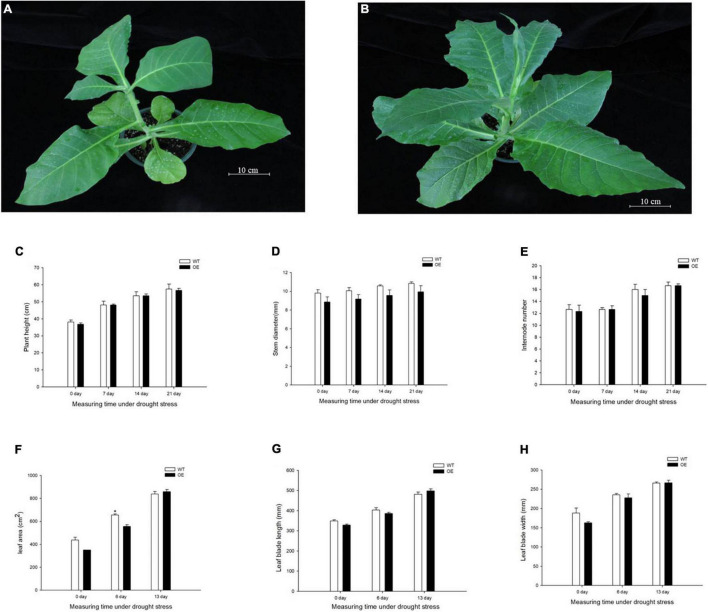
*PpCCoAOMT* did not affect plant height, stem diameter, or the number of internodes, although it decreased leaf growth in transgenic tobacco plants under transient drought. **(A)** Phenotype of the WT line after recovery treatment. **(B)** Phenotype of the *PpCCoAOMT*-overexpressing (OE) line after recovery treatment. **(C)** Plant height, **(D)** stem diameter, **(E)** internode number, **(F)** leaf area, **(G)** leaf length, and **(H)** leaf width of the WT and OE lines under drought stress. Data are means (± SDs) of three replicates. Significant differences between WT and OE lines were determined using independent samples *t-*tests. *P < 0.05, **P < 0.01.

**FIGURE 3:**
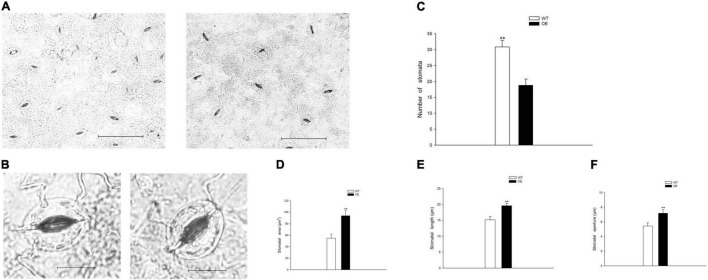
*PpCCoAOMT* overexpression reduces stomatal number but increases stomatal area, length, and aperture under normal conditions. **(A)** Advanced upright optical microscopic images of the WT and OE lines. Bars = 100 μm. **(B)** Image of a single stoma from the WT and OE transgenic lines. Bars = 20 μm. Images were acquired using a BX51 bright-field microscope with a DP70 charge-coupled device camera (Olympus, Tokyo, Japan). **(C)** The number of stomata was calculated for each line using Image-Pro Plus 6.0 software. Data are means (± SDs) of 25 replicates. **(D)** Stomatal area, **(E)** stomatal length, and **(F)** stomatal aperture of the WT and OE transgenic tobacco lines were determined using Image-Pro Plus 6.0 software. Data are means (± SDs) of 35 replicates. A microscopic ruler (Olympus) was used to calibrate size. Significant differences between WT and OE lines were determined using independent samples *t-*tests. *P < 0.05, **P < 0.01.

To evaluate the growth status of transgenic tobacco, we measured plant height, stem diameter, and the number of internodes under the normal watered and drought treatments. The transgenic tobacco plants and the WT controls after recovery treatment are shown in Figures 2A,B. The plant height, stem diameter, and number of nodes were not significantly different after the 21-day drought (Figures 2C–E). Despite the lack of significant differences in plant growth between the WT and OE lines, the leaf area was significantly smaller in OE plants than in WT plants after 6 days without irrigation. In addition, the leaf area of the OE tobacco plants rapidly increased after 13 days of drought, in contrast to leaf area in WT plants (Figure 2F). The leaf area is proportional to the leaf length and leaf width, but the decreased leaf area was not accompanied by a significant decrease in leaf length or leaf width compared to WT plants (Figures 2G,H). It has been widely reported that smaller leaves of transgenic plants maintain reduced transpiration because of smaller stomata with reduced stomatal pores (Nir et al., 2014; Zhong et al., 2014; Illouz-Eliaz et al., 2020). We examined the abaxial leaf epidermal tissues *via* microscopy. The number of stomata in the OE transgenic tobacco significantly decreased to 60.88% of the number in WT plants (Figures 3A,C). Additionally, microscopy analyses revealed larger stomata in OE plants (Figure 3B). The stomatal area, length, and aperture in the OE lines under normal conditions increased by 171.61, 129.10, and 132.54%, respectively, compared to values in WT plants (Figures 3D–F). Taken together, the overexpression of *PpCCoAOMT* decreased leaf area and reduced the number of stomata after cessation of watering for 6 days, but increased stomatal area, length, and aperture under normal conditions.

**FIGURE 4:**
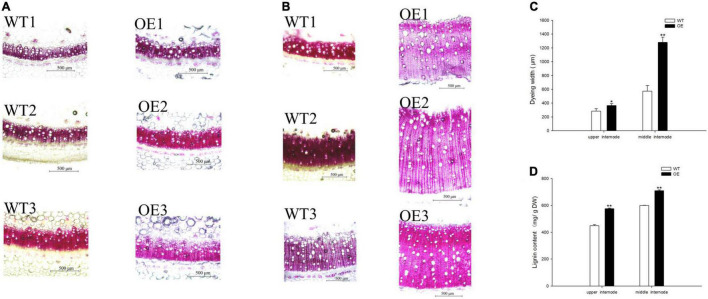
*PpCCoAOMT* overexpression increases xylem vessel proliferation and expansion. **(A)** Representative upper internode cross-sections of WT and OE plants stained with Wiesner stain. Bars = 500 μm. **(B)** Representative middle internode cross-sections of WT and OE plants stained with Wiesner stain. Bars = 500 μm. **(C)** The dye widths of the WT and OE transgenic tobacco lines were determined using Image-Pro Plus 6.0 software. Data are means (± SDs) of nine replicates. Each replicate was measured three times at different angles. **(D)** Lignin content in WT and OE plants. Data are means (± SDs) of triplicates Significant differences between WT and OE lines were determined using independent samples *t-*tests. *P < 0.05, **P < 0.01.

Lignin staining and content were examined because CCoAOMT is an important enzyme that participates in lignin monomer synthesis (Yang et al., 2017; Xiao et al., 2020; Zhao et al., 2021). The dye width in the OE line significantly increased (Figures 4A,B), such that it was 28.18 and 124.24% wider than in WT plants at the upper and middle internodes, respectively (Figure 4C). Moreover, quantitative analysis of lignin content showed a similar trend in which the OE line increased to 128.46 and 118.43% of the content in WT plants (Figure 4D). These results demonstrated that the upregulation of *PpCCoAOMT* promotes lignin biosynthesis.

### Overexpression of *PpCCoAOMT* Increases Drought Stress Tolerance

**FIGURE 5:**
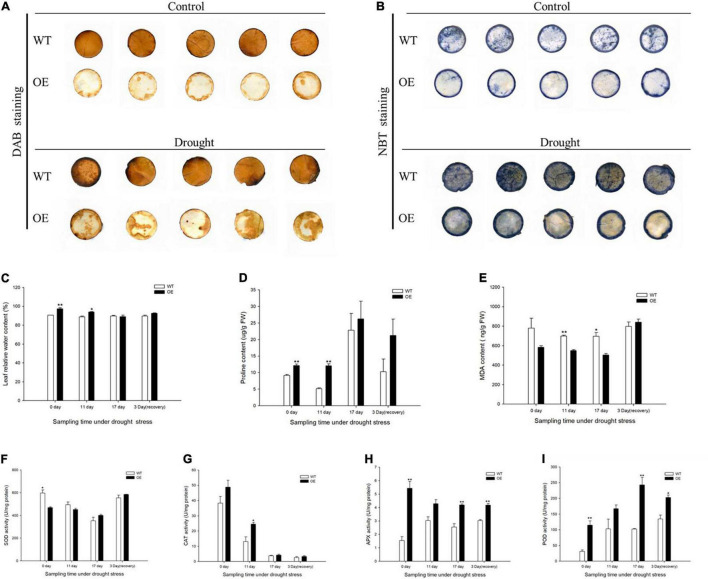
Overexpression of *PpCCoAOMT* promotes tolerance to drought stress. **(A)** Diaminobenzidine (DAB) (displaying H_2_O_2_) staining of leaves from WT and OE seedlings under normal and drought stress treatment. **(B)** Nitro blue tetrazolium (NBT) (displaying O_2_^–^) staining of WT and OE leaves under normal and drought conditions. **(C)** Mean leaf relative water content (RWC), **(D)** proline content, **(E)** malondialdehyde (MDA) content, and **(F)** superoxide dismutase (SOD), **(G)** catalase (CAT), **(H)** ascorbate peroxidase (APX), and **(I)** peroxidase (POD) activities in WT and OE plants during drought stress and recovery periods. Data are means (± SDs) of triplicates. Significant differences between WT and OE lines were determined using independent samples *t-*tests. *P < 0.05, **P < 0.01.

The production of H_2_O_2_ was monitored by DAB staining, which yields a brown precipitate produced by the peroxidase-catalyzed reaction of the dye with H_2_O_2_. WT plants accumulated more H_2_O_2_ in leaves under normal and drought treatments. O_2_^–^ accumulation was demonstrated by a blue NBT precipitate, with a substantially decreased extent of NBT staining in the OE transgenic tobacco leaves, indicating that overexpression of the PpCCoAOMT gene led to less impairment under drought stress (Figures 5A,B). Related physiological indices were measured to further evaluate the growth statuses of WT and OE lines under drought. The leaf RWC was significantly higher in OE plants than in WT plants under normal conditions (Figure 5C). Thus, we speculate that the number of stomata was a major factor affecting transpiration. Eleven days into the drought treatment, leaf RWC decreased in the OE lines but remained significantly higher than in WT plants (Figure 5C). Proline content showed a similar pattern, such that OE plants were significantly different from WT plants on days 0 and 11. When measured at day 17, proline content was 14.98% greater in OE plants than in WT plants. Although proline content decreased after 3 days of rehydration, the OE tobacco plants retained more proline than did the WT plants (Figure 5D). The MDA content, an essential indicator of membrane injury, exhibited greater accumulation in WT plants than in OE plants during the drought stress (Figure 5E).

Antioxidant enzyme activities were determined, including superoxide dismutase (SOD), catalase (CAT), ascorbate peroxidase (APX), and peroxidase (POD). SOD activity was initially lower in OE plants than in WT plants, but its was greater in OE plants on day 17 and 3 days of recovery (Figure 5F). CAT, APX, and POD activities were higher in the OE line than in WT plants at all sampling times. CAT activity was significantly greater (by 86.75%) in the OE line than in WT plants after 11 days without irrigation (Figure 5G). Because SOD and CAT were the first two responding antioxidant enzymes, they managed severe ROS damage during the prolonged water deficit. APX and POD activities significantly increased in the OE transgenic plants in response to 17 days of drought stress; they were 64.71 and 137.33% greater than activities in WT plants. APX and POD activities in OE lines were also higher during recovery, such that they increased by approximately 1.4- and 1.5-fold, respectively, compared with the WT (Figures 5H,I). These results indicated that OE-*PpCCoAOMT* plants were more resistant to drought stress because of higher RWC, lower MDA, and the accumulation of proline and antioxidant enzyme activities.

### Comparison of Metabolite Profiles Between Wild-Type and OE-*PpCCoAOMT* Plants

**FIGURE 6:**
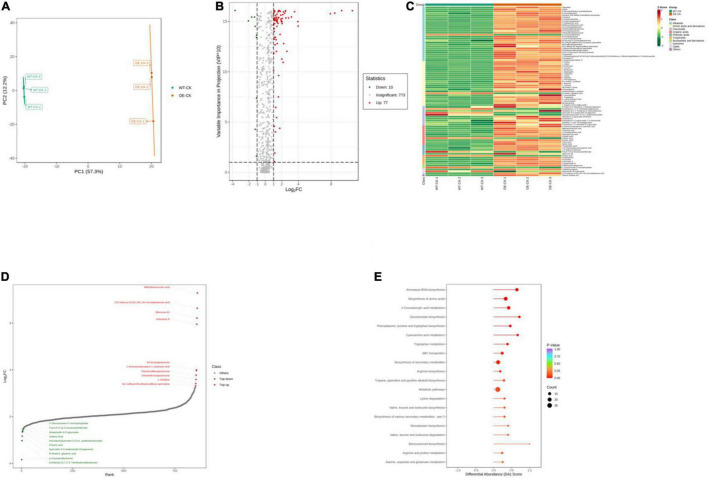
Qualitative and quantitative analyses of differentially expressed metabolites in WT and OE plants. **(A)** PCA score plots for the mass spectra data of WT and OE samples. The abscissa represents the first principal component (PC1), while the ordinate represents the second principal component (PC2). **(B)** Volcano plot of differentially expressed metabolites between WT and OE plants. The abscissa represents the contents of differentially expressed metabolites between the two samples; a greater absolute value of the abscissa indicates a greater difference in relative content. A larger ordinate value indicates a more credible difference. Red scatter indicates metabolites that were significantly upregulated; green scatter indicates metabolites that were significantly downregulated; gray scatter indicates metabolites with no significant difference. **(C)** Clustering analysis of the metabolome data from WT and OE plants. Heatmap representation of the levels of differentially expressed metabolites and the color indicated low (green) to high (red). Each line in the heatmap represents a metabolite. Ten classes were evident after the clustering analysis. **(D)** The top 10 upregulated and downregulated metabolites between WT and OE plants. Red dots represent upregulated metabolites; green dots represent downregulated metabolites. **(E)** KEGG enrichment analysis of differentially accumulating metabolites between WT and OE samples. DA score represents the overall changes in all metabolites in the metabolic pathways; a score of 1 indicates upregulated trend, while a score of –1 indicates a downregulated trend. Each bubble in the plot represents the number of associated metabolites; *P-*values are indicated by colors.

We performed ultrahigh performance liquid chromatography-electrospray ionization-tandem mass spectroscopy to gain insight into the underlying metabolite profiles of OE-*PpCCoAOMT* and WT plants. Mass spectral data were processed using Analyst 1.6.3 software. The data were subjected to PCA; the score plot showed strong and significant separation between the OE and WT plants along the first component (Figure 6A). To identify differentially expressed metabolites in OE and WT plants, metabolites were selected with a fold-change ≥ 2 (upregulated) or ≤ 0.5 (downregulated), and a variable importance of projection value ≥ 1 from the orthogonal projections to latent structures discriminant analysis model. In this study, 87 differentially expressed metabolites were identified including 77 upregulated (red scatter points) and 10 downregulated (green scatter points) (Figure 6B). These 87 metabolites were categorized into 10 classes, including 28 alkaloids, 23 amino acids, and derivatives, 10 flavonoids, 10 organic acids, 5 phenolic acids, 5 terpenoids, 2 nucleotides and derivatives, 2 quinones, 1 lipid, and 1 other; unit variance scaling of peak areas was then applied to perform hierarchical cluster analysis (Supplementary Table 1 and Figure 6C). Among all metabolites, the top 10 upregulated metabolites were alkaloids, organic acids, and flavonoids, while the top 10 downregulated metabolites were flavonoids and amino acids and derivatives (Figure 6D).

Complex metabolic reactions and regulation in living organisms usually involve different genes and proteins that form complex pathways and networks, which interact with and regulate each other; these exchanges result in systematic changes in the metabolome (Töpfer et al., 2015). Accordingly, we obtained the detailed pathways of the differentially expressed metabolites based on the KEGG database (Supplementary Table 2). In total, 51 pathways were involved in WT plants vs. OE plants; the top 20 most significantly enriched pathways are presented in a bubble plot (Figure 6E). Considering the DA score, *P*-value, and the number of enriched metabolites, the top 5 pathways were “Glucosinolate biosynthesis,” “Cyanoamino acid metabolism,” “Aminoacyl-tRNA biosynthesis,” “Phenylalanine, tyrosine, and tryptophan biosynthesis,” and “2-Oxocarboxylic acid metabolism.” All of these metabolic pathways showed a *P*-value < 0.01 in the enrichment analysis and were upregulated (Figure 6E).

### Variations in the Metabolites From the Wild-Type and OE-*PpCCoAOMT* Plants Under Drought Stress

**FIGURE 7:**
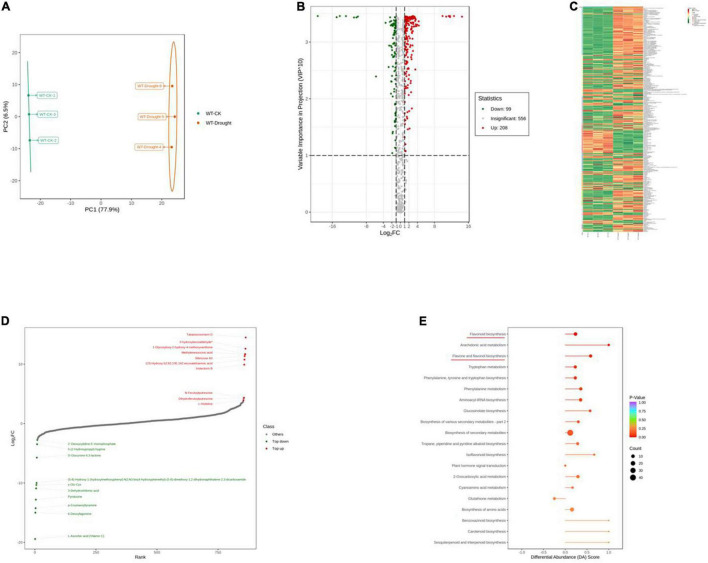
Qualitative and quantitative analyses of differentially expressed metabolites in WT plants after drought stress. **(A)** PCA score plots for the mass spectra data of WT and OE samples. The abscissa represents the first principal component (PC1), while the ordinate represents the second principal component (PC2). **(B)** Volcano plot of differentially expressed metabolites between WT and OE plants. The abscissa represents the contents of differentially expressed metabolites between the two samples; a greater absolute value of the abscissa indicates a greater difference in relative content. A larger ordinate value indicates a more credible difference. Red scatter indicates metabolites that were significantly upregulated; green scatter indicates metabolites that were significantly downregulated; gray scatter indicates metabolites with no significant difference. **(C)** Clustering analysis of the metabolome data from WT and OE plants. Heatmap representation of the levels of differentially expressed metabolites and the color indicated low (green) to high (red). Each line in the heatmap represents a metabolite. Ten classes were evident after the clustering analysis. **(D)** The top 10 upregulated and downregulated metabolites between WT and OE plants. Red dots represent upregulated metabolites; green dots represent downregulated metabolites. **(E)** KEGG enrichment analysis of differentially accumulating metabolites between WT and OE samples. DA score represents the overall changes in all metabolites in the metabolic pathways; a score of 1 indicates upregulated trend, while a score of –1 indicates a downregulated trend. Each bubble in the plot represents the number of associated metabolites; *P-*values are indicated by colors.

**FIGURE 8:**
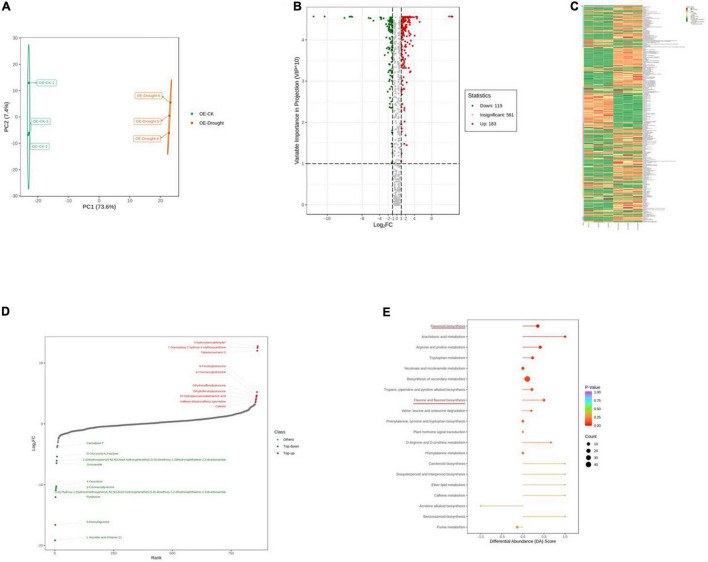
Qualitative and quantitative analyses of differentially expressed metabolites in OE plants after drought stress. **(A)** PCA score plots for the mass spectra data of WT and OE samples. The abscissa represents the first principal component (PC1), while the ordinate represents the second principal component (PC2). **(B)** Volcano plot of differentially expressed metabolites between WT and OE plants. The abscissa represents the contents of differentially expressed metabolites between the two samples; a greater absolute value of the abscissa indicates a greater difference in relative content. A larger ordinate value indicates a more credible difference. Red scatter indicates metabolites that were significantly upregulated; green scatter indicates metabolites that were significantly downregulated; gray scatter indicates metabolites with no significant difference. **(C)** Clustering analysis of the metabolome data from WT and OE plants. Heatmap representation of the levels of differentially expressed metabolites and the color indicated low (green) to high (red). Each line in the heatmap represents a metabolite. Ten classes were evident after the clustering analysis. **(D)** The top 10 upregulated and downregulated metabolites between WT and OE plants. Red dots represent upregulated metabolites; green dots represent downregulated metabolites. **(E)** KEGG enrichment analysis of differentially accumulating metabolites between WT and OE samples. DA score represents the overall changes in all metabolites in the metabolic pathways; a score of 1 indicates upregulated trend, while a score of –1 indicates a downregulated trend. Each bubble in the plot represents the number of associated metabolites; *P-*values are indicated by colors.

To further explore the major physiological changes caused by water deficit in the WT and OE lines, the metabolic profiles were compared to verify metabolite fluctuations during drought treatment. PCA plots showed that the cumulative contribution rates reached 84.4 and 81.0% from the intra-group comparison of the WT-CK vs. the WT-Drought and the OE-CK vs. OE-Drought samples, respectively. In total, WT-CK, WT-Drought, OE-CK, and OE-Drought were significantly separated, whereas the repeated samples from each group were clustered together (Figures 7A, 8A). The metabolites were screened by combining the variable importance of projection value and the fold-change value. We identified 307 and 302 differentially expressed metabolites in the WT and OE groups. Overall, 208 metabolites increased and 99 decreased in the WT-CK compared with their counterparts in WT-Drought plants. There were 183 upregulated and 119 downregulated metabolites between the OE-CK and OE-Drought plants (Figures 7B, 8B). According to the results of hierarchical cluster analysis, the differentially expressed metabolites of each group were visualized in thermogram format; flavonoids constituted the largest class in the WT group, whereas alkaloids constituted the largest class in the OE group (Figures 7C, 8C). In the WT group, 64 of 65 (98.46%) flavonoids were upregulated, whereas 49 of 54 (90.74%) flavonoids were upregulated in the OE group. For alkaloids, 36 of 56 were upregulated (64.29%) and 20 of 56 (35.71%) were downregulated in the WT group. In comparison, 38 of 58 (65.52%) increased and 20 of 58 (34.48%) decreased in the OE-CK vs. OE-Drought comparison. Notably, 90% (45 of 50) and 86.84% (33 of 38) of lipids were downregulated in the OE and WT groups, respectively (Supplementary Tables 3, 5). We then investigated the top 10 differentially expressed metabolites. Alkaloids and flavonoids exhibited the greatest increases. Unexpectedly, alkaloids also exhibited the greatest increase in the WT-CK vs. WT-Drought group comparison. Most differentially expressed metabolites in the OE-CK vs. OE-Drought samples were alkaloids (Figures 7D, 8D).

Moreover, we mapped the differentially expressed metabolites to the KEGG database and analyzed the pathway information. The results showed that the WT group involved 67 pathways, whereas the OE-CK and OE-Drought plants involved 74 pathways (Supplementary Tables 4, 6). The major pathways are presented in bubble plots. The top 5 pathways in the WT-CK vs. WT-Drought comparison (Figure 7E) were “Arachidonic acid metabolism,” “Flavonoid biosynthesis,” “Flavone and flavonol biosynthesis,” “Tryptophan metabolism,” and “Phenylalanine, tyrosine, and tryptophan biosynthesis.” The top 5 pathways in the OE-CK vs. OE-Drought group comparison (Figure 8E) were “Arachidonic acid metabolism,” “Flavonoid biosynthesis,” “Tryptophan metabolism,” “Arginine and proline metabolism,” and “Flavone and flavonol biosynthesis.” In addition, “Flavonoid biosynthesis” and “Flavone and flavonol biosynthesis” were two specialized pathways that were enriched and significantly different (*P* < 0.05) in the two groups, indicating that flavonoids may be closely related to drought tolerance.

### Unique and Common Drought-Responsive Differential Accumulated Metabolites Between Wild-Type and OE-*PpCCoAOMT* Plants

**FIGURE 9:**
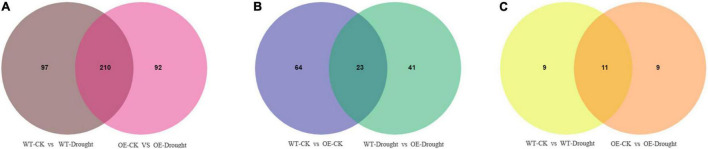
Venn diagrams of differentially accumulated metabolites (DAMs). **(A)** Venn diagram showing common DAMs between normal and drought conditions in WT and OE plants. **(B)** Venn diagram showing unique and common DAMs between WT and OE plants under normal and drought conditions. **(C)** Venn diagram showing common DAMs in the top 20 differentially expressed metabolites in WT and OE plants after drought stress.

A Venn diagram was constructed to examine the unique and common differentially expressed metabolites between the drought treatment and unstressed control and between the WT and OE plants after drought treatment. Large numbers of differential accumulated metabolites (DAMs) were identified in WT and OE plants. In total, 307 and 302 were DAMs in WT and OE plants, respectively, among which 210 DAMs were common drought-responsive metabolites. Moreover, 139 of the 210 metabolites were upregulated and 69 were downregulated in WT and OE plants under the drought treatment (Figure 9A and Supplementary Table 7). In addition, among the top 20 DAMs responding to drought in WT and OE plants, 11 metabolites were shared, demonstrating a shared drought response mechanism (Figure 9C). Sixty-four and 41 DAMs were specifically found in WT vs. OE plants under normal and drought conditions. Overall, 23 DAMs were common compounds, indicating that the essential difference between the WT and OE lines was unrelated to drought. Seventeen of the 23 metabolites were upregulated, while only one was downregulated in WT vs. OE plants regardless of the drought treatment (Figure 9B and Supplementary Table 8).

**FIGURE 10:**
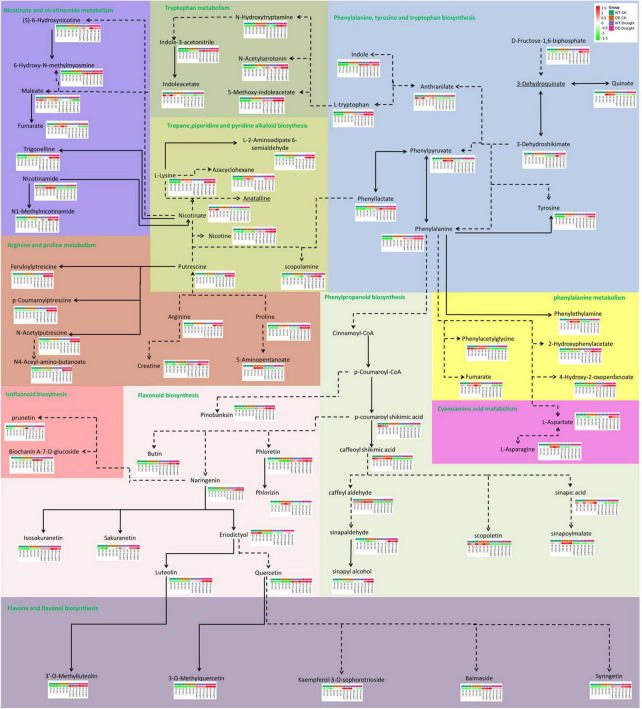
Analysis of pathway metabolite changes between WT and OE plants under normal and drought treatments. Dashed arrows represent multiple enzymatic steps. Red represents upregulated metabolites, while green represents downregulated metabolites.

The total number of metabolites in the plant kingdom is estimated to exceed 200,000 (Schauer and Fernie, 2006; Le Roy et al., 2016). To cope with abiotic stress, plants have evolved mechanisms to regulate the levels of metabolites, such as phenylpropanoids and alkaloids. The DAMs between WT and OE plants under normal and drought treatments were annotated in the metabolic pathways (Figure 10). In total, 69 DAMs related to 10 pathways (including core and specialized metabolism) were shared, demonstrating a role for the *PpCCoAOMT* response pathway in the drought metabolome. The results of KEGG enrichment analysis showed that most DAMs were annotated in flavonoid biosynthesis; flavone and flavonol biosynthesis were upregulated, while phenylpropanoid biosynthesis was downregulated, after drought treatment in WT and OE plants. Notably, the arginine and proline metabolic pathway increased in OE-Drought plants.

## Discussion

### *PpCCoAOMT* Contributes to Drought Tolerance in Transgenic Tobacco

In this study, we demonstrated that transgenic tobacco plants overexpressing the *PpCCoAOMT* gene, which codes for lignin biosynthesis, were better able to cope with drought stress than were non-transformed control plants. This conclusion was supported by the positive RWC, proline content, and antioxidant enzyme values, as well as the decreases in MDA and ROS contents, including O_2_^–^ and H_2_O_2_, observed in the leaves of transgenic plants after 17 days of water deficit. A plant’s first response, when subjected to a water shortage, is to avoid low water potential by decreasing stomatal conductance; it also prevents water loss by hardening the cell wall or promoting water influx through osmotic adjustments. The presence of significantly higher leaf RWC on days 0 and 11 after the drought treatment in transgenic tobacco (Figure 5C) may have occurred because of smaller leaf area (Figure 2F) and lower stomatal number (Figures 3A,C), despite increased stomatal area (Figures 3B,D). Stomatal density reportedly exhibits a negative correlation with stomatal size (Yu et al., 2013; Wang et al., 2020). We also detected this correlation in our study, such that stomatal length, aperture, and area were significantly higher in the OE tobacco lines than in WT plants, whereas the number of stomata was lower in the OE plants. Therefore, we propose that the total number of stomata was the major factor that affected stomatal conductance, which brought higher leaf RWC in the OE line under the transient drought condition. Moreover, with the prolonged drought period, additional tolerance mechanisms (e.g., hardening of the cell wall and osmotic adjustment) confer protection against low water potential and help to avoid cell dehydration (Verslues et al., 2006). Thus far, many studies have reported that regulation of the *PpCCoAOMT* gene alters lignin composition and content. The downregulation (Pinçon et al., 2001; Boerjan et al., 2003; Wagner et al., 2011; Li et al., 2013; Pang et al., 2014; Wang et al., 2017) or upregulation (Zhang et al., 2014; Zhang Y. et al., 2019; Yang et al., 2017; Fu et al., 2019, 2020; Zhao et al., 2021) of *CCoAOMT* leads to a decrease or increase of lignin content in transgenic plants, respectively. In this study, the OE lines had significantly accumulated lignin deposits under normal conditions (Figures 4A,C), a conclusion supported by a higher lignin content in the upper and middle internodes (Figure 4D). Because lignin is covalently linked to non-cellulosic polysaccharides, the reinforcement of plant walls facilitates defense against abiotic assaults (Boudet, 2007). With a strengthened plant cell wall, plants can maintain water resistance to prolonged drought. Osmotic adjustments are another method to maintain osmolality; proline is an osmotic adjustment mediator (Zhang et al., 1999) and a ROS scavenger that stabilizes DNA, proteins, and membranes (Alia et al., 2001). The accumulation of proline was confirmed in OE-*PpCCoAOMT* plants under normal and drought conditions (Figure 5D), indicating that the overexpression of *PpCCoAOMT* in transgenic plants contributed to drought tolerance.

Stomatal closure is one of the earliest responses to drought; it has an important role in water loss control in plants (Chaves et al., 2003; Wang et al., 2020), which subsequently downregulates CO_2_ uptake and causes a decline in photosynthesis. Thus, the electron transport chain becomes over-reduced and favors excess ROS production. In plants, MDA is an indicator of cell membrane damage caused by ROS-induced lipid peroxidation (Zhong et al., 2014). The OE plants had significantly lower MDA content than did the WT plants (Figure 5E). Moreover, H_2_O_2_ and O_2_^–^ staining (Figures 5A,B) revealed that *PpCCoAOMT* mitigated the effects of the water deficit. These observations suggested activation of the antioxidant system. The activities of the four enzymes evaluated in this study were higher because of the high free proline concentration present in the leaves of transgenic plants. However, the APX and POD (Figures 5H,I) activities significantly increased in OE tobacco, even under the control treatment, possibly because POD is a key enzyme involved in monolignol polymerization in the secondary cell wall (Bonawitz and Chapple, 2010; Miao and Liu, 2010; Liu et al., 2011; Alejandro et al., 2012). Because of higher lignin content in OE-*PpCCoAOMT* plants, it is perhaps unsurprising that POD activity was approximately 3.6-fold greater in OE plants than in WT plants on day 0. APX enhances the tolerance of transgenic Arabidopsis by increasing the accumulation of lignin and maintaining the level of H_2_O_2_ (Shafi et al., 2015). APX, POD, and CAT are involved in scavenging H_2_O_2_ to H_2_O and O_2_ (Mittler, 2002). Thus, the decrease in CAT (Figure 5G) activity during drought stress may orchestrate the regulation of these three enzymes to ensure balance with other metabolic processes. This balancing also occurs in *P5CSF129A*-overexpressing “Swingle” citrumelos and *P5CR*-overexpressing soybeans (Kocsy et al., 2005; de Campos et al., 2011).

### *PpCCoAOMT* Affects Primary and Specialized Metabolism, Leading to Flavonoid Accumulation Under Normal and Drought Conditions

In general, H_2_O_2_ is both a stress-induced ROS and an important regulatory component in lignin biosynthesis (Kováčik et al., 2010; Liu et al., 2015; Xia et al., 2018). It is unknown whether *PpCCoAOMT* contributes to crosstalk between the lignin biosynthetic pathway and the abiotic stress response. Thus, we used OE-*PpCCoAOMT* transgenic tobacco plants to analyze widely targeted metabolomes under normal and drought conditions. Among the top 10 ranked DAMs for the WT-CK vs. OE-CK group comparison (Figure 6D and Supplementary Table 1), two upregulated and three downregulated metabolites were flavonoids, three upregulated and one downregulated metabolite were alkaloids, and two upregulated and one downregulated metabolite were organic acids. In the WT-CK vs. WT-Drought group comparison (Figure 7D and Supplementary Table 3), two upregulated and four downregulated metabolites were alkaloids, two upregulated metabolites were flavonoids, and one upregulated and one downregulated metabolite were organic acids. In the OE-CK vs. OE-Drought group comparison (Figure 8D and Supplementary Table 5), four upregulated and four downregulated metabolites were alkaloids. These results indicate that flavonoids and organic acids changed in OE-*PpCCoAOMT* transgenic tobacco plants under normal conditions, which may have begun acting in ROS scavenging during the initial stages of water deprivation (Nakabayashi and Saito, 2015; Jiang et al., 2016; Khan et al., 2020); this may have prevented further cell damage during prolonged stress. As mentioned above, hierarchical cluster analysis (Figure 6C) results supported the conclusion that *PpCCoAOMT* modulated flavonoids and organic acids in the watered plants.

Metabolic pathway analysis of the DAMs revealed that the top 5 pathways for the WT-CK vs. OE-CK group comparison were mostly involved in primary metabolism (Figure 6E). However, in WT (Figure 7E) and OE (Figure 8E) plants, the drought treatment affected the KEGG pathways that were enriched in primary and specialized metabolism, in which WT plants showed more secondary metabolism. This observation is consistent with the hierarchical cluster analysis of the WT (Figure 7C) and OE (Figure 8C) plants, whereby 65 flavonoids and 56 alkaloids differentially accumulated in WT samples after drought treatment compared to 58 flavonoids and 54 alkaloids in the OE-*PpCCoAOMT* transgenic tobacco.

CCoAOMT is a key enzyme involved in the first methylation step required to produce lignin monomers in the phenylpropanoid metabolic pathway. The phenylpropanoid metabolic pathway is on the boundary of core metabolism (traditionally known as “primary metabolism”) and specialized metabolism (traditionally known as “secondary metabolism”) and directs the metabolic flux from the core to specialized metabolism (Fraser and Chapple, 2011; Dong and Lin, 2021). In OE-*PpCCoAOMT* transgenic tobacco plants, metabolic flux redirection within the primary metabolism (Figure 10) led to large quantities of proline accumulation in plant tissues in response to different abiotic stressors during experiments involving exogenous application (Hoque et al., 2007a,b, 2008; Ozden et al., 2009) or genetic manipulation (Kocsy et al., 2005; Molinari et al., 2007); this redirection also caused decreases in the contents of nicotinate involved in drought tolerance and biomass (Ahmad et al., 2021). The metabolic fluxes of the WT and OE plants were redirected to the flavonoid biosynthetic pathway with significantly different concentrations after drought treatment (Figure 10). In particular, quercetin has an important role in the drought tolerance of tea plants (Sun J. et al., 2020), *Holcus lanatus*, and *Alopecurus pratensis* (Gargallo-Garriga et al., 2015). Luteolin may have antioxidant activity at low concentrations (Singh et al., 2014). Treatment with naringenin reduces oxidative damage and enhances tolerance to osmotic stress (Yildiztugay et al., 2020).

## Conclusion

In conclusion, we have characterized the lignin biosynthetic gene, *PpCCoAOMT*, which contributes to lignin deposition and flavonoid accumulation; it has important roles in controlling drought tolerance, stomatal number, and aperture size. There was no cost to the growth of transgenic plants (Zhang Y. et al., 2019) based on leaf size (Figures 2A,B,F), plant height (Figure 2C), or stem diameter (Figure 2D), which highlights the importance of the *PpCCoAOMT* gene in future attempts to breed drought-resistant plants.

## Data Availability Statement

The datasets presented in this study can be found in online repositories. The names of the repository/repositories and accession number(s) can be found in the article/Supplementary Material.

## Author Contributions

J-LS and Z-YW completed most research experiments. Y-HW, JD, and C-YW contributed to data analysis. X-QZ, SC, and X-LH helped with manuscript reviewing. X-MX and T-XZ revised the manuscript. All authors read and approved the final manuscript.

## Conflict of Interest

The authors declare that the research was conducted in the absence of any commercial or financial relationships that could be construed as a potential conflict of interest.

## Publisher’s Note

All claims expressed in this article are solely those of the authors and do not necessarily represent those of their affiliated organizations, or those of the publisher, the editors and the reviewers. Any product that may be evaluated in this article, or claim that may be made by its manufacturer, is not guaranteed or endorsed by the publisher.

## Footnotes

1
www.shimadzu.com.cn/

2
www.appliedbiosystems.com.cn/

3
http://www.kegg.jp/kegg/compound/

4
http://www.kegg.jp/kegg/pathway.html
