# Supplementary material for: Overexpression of Pennisetum purpureum CCoAOMT Contributes to Lignin Deposition and Drought Tolerance by Promoting the Accumulation of Flavonoids in Transgenic Tobacco
Source: Front Plant Sci. 2022 May 10;13:884456. doi: 10.3389/fpls.2022.884456 (PMC9129916; doi:10.3389/fpls.2022.884456)
Supplement: Supplementary file 9 [file Data_Sheet_1.PDF]

Supplementary data

Fig. S1 Elephant grass (*Pennisetum purpureum*) CCoAOMT genomic clone structure. Blue boxes represent exons, whereas green boxes represent introns; black solid lines indicate the promoter region of 349 bp and the 3’ untranslated region (UTR) of 184 bp, respectively.

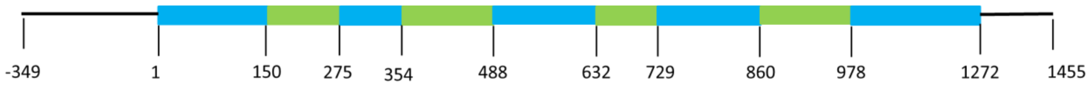

Fig. S2 Phylogenetic tree for the PpCCoAOMT predicted protein and orthologs from gymnosperms, dicots, and monocots. The neighbor-joining (NJ) method was used to establish evolutionary relationships. The bootstrap consensus tree was inferred from 500 replicates and the numeric values at the branches represent bootstraps. The ruler in the lower left corner indicates the genetic distance.

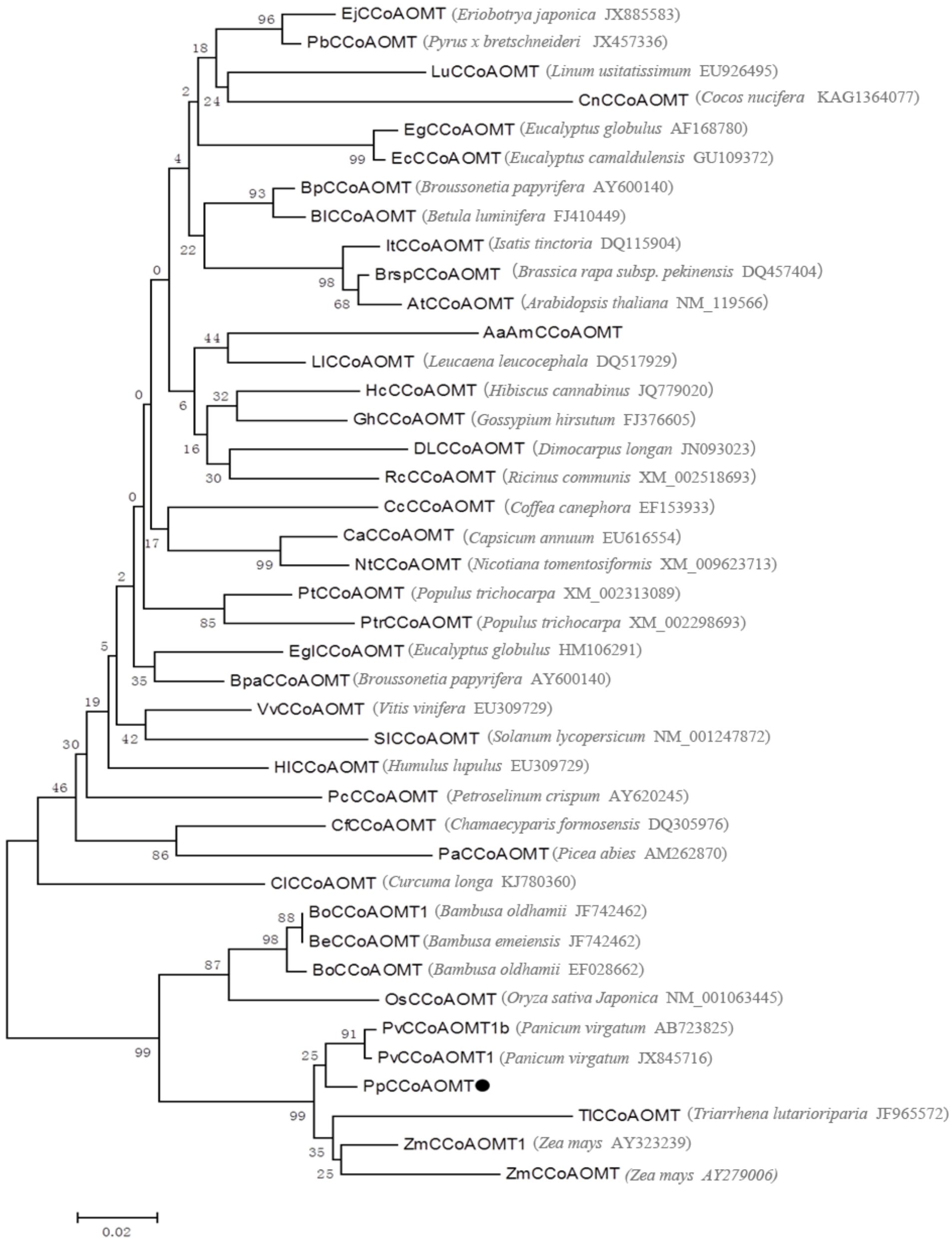

Fig. S3 PCR and RT-PCR analyses of OE-*PpCCoAOMT* transgenic plants. (A) PCR tests of partial OE-*PpCCoAOMT* transgenic plants with the selectable marker gene. Bar, a tobacco (*Nicotiana tabacum*) internal reference gene actin served as the template loading control. M, marker D2000; -, Lanes 1-4, DNA from WT plants was used as the template; Lanes 5-16, DNA from transgenic plants was used as the template; (B) RT-PCR of the specific *PpCCoAOMT* gene in OE-*PpCCoAOMT* transgenic plants; the tobacco (*N. tabacum*) internal reference gene actin served as the template loading control. M, marker D2000; Lanes 1-12, cDNA from transgenic plants was used as the template. Lanes 13-16, cDNA from WT plants was used as the template.

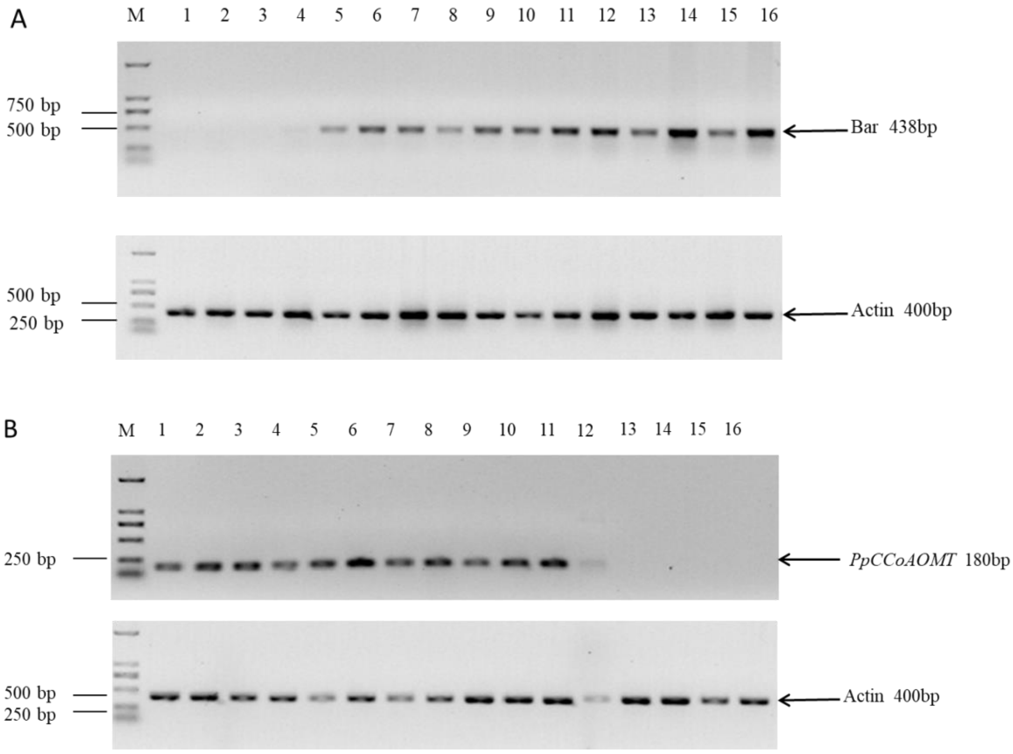

A
M 1 2 3 4 5 6 7 8 9 10 11 12 13 14 15 16
750 bp
500 bp
Bar 438bp
500 bp
250 bp
Actin 400bp
B
M 1 2 3 4 5 6 7 8 9 10 11 12 13 14 15 16
250 bp
PpCCoAOMT 180bp
500 bp
250 bp
Actin 400bp
